# mTOR and its link to the picture of Dorian Gray - re-activation of mTOR promotes aging

**DOI:** 10.18632/aging.100240

**Published:** 2010-12-08

**Authors:** Józefa Węsierska-Gądek

**Affiliations:** Cell Cycle Regulation Group, Div.: Institute of Cancer Research, Department of Medicine, Comprehensive Cancer Center, Medical University of Vienna, Vienna, Austria

The limited dividing potential of normal cells leads to replicative cellular senescence that is defined by irreversible loss of proliferative potential, adoption of characteristic morphology and expression of typical biomarkers [1,2]. Cellular senescence acts as a barrier to malignant cell transformation *in vivo* [3] and may contribute to organismal ageing [1,2,4]. The p53 tumor suppressor is a major determinant of cellular senescence [5]. It plays a crucial role in the integration of stress signaling and coordination of cellular responses to stress. Depending on the kind of stress stimuli, stress strength and cellular context, activation of tumor suppressor p53 can induce reversible quiescence, cellular senescence and apoptosis [6]. The function of wt p53 is extremely complex and, of course, has to be explained within the context of the expression of distinct p53 isoforms, levels of p53-induced micro-RNA and a complex network of p53-interacting proteins. Two p53 isoforms (Δ133p53 and p53β), and in particular their mutual interaction, seem to act as endogenous regulators of cellular senescence in normal human fibroblasts [7]. The relevance of these findings to *in vivo* events reflects the fact that increases in Δ133p53 expression and reductions in p53β expression have been observed *in vivo* in colon adenomas with senescent phenotypes [8].

The cancer-protective function of wt p53 is also related to its master function in the regulation of various stages of apoptosis [9]. However, wt p53 plays a dual role in the control of cells' suicide; depending on stress strength it can either prevent or induce programmed cell death. While the apoptosis-promoting function of p53tumor suppressor protein has been intensively scrutinized and is indisputable, the pro-survival function of wt p53 (mediated *inter alia* via TIGAR, its downstream target) has been less explored and due to some controversies remains an object of debate. However, p53 tumor suppressor has been shown to respond to metabolic changes and to influence metabolic pathways through several mechanisms. In response to a lack of nutrients, p53 becomes activated through the activation of AMP-activated protein kinase (AMPK) and the inhibition of AKT. p53 protein further induces AMPK (both directly and indirectly through the sestrins) and activates the expression of tuberous sclerosis 2 (TSC2), resulting in the inhibition of mTOR, a cytoplamic kinase that transfers signals induced by growth factors to cellular machinery promoting cell proliferation and survival.

However, cross-talk between p53 and the mTOR signaling pathways is more complex. Recent studies provide evidence regarding the decision-making role of these pathways in the choice between p53-mediated cellular quiescence and senescence. The paradoxical function of p53, on the one hand repressing cellular senescence by promoting quiescence, and on the other inducing senescence, has been systematically addressed and rigorously studied by M. Blagosklonny and colleagues [10-11]. Demidenko et al. [10] have shown that suppression of cellular senescence by wt p53 is associated with p53-induced quiescence and requires p53 transactivation and inhibition of mTOR. Conversely, the activation of mTOR by depletion of TSC2, a negative regulator of mTOR, favors senescence in normal cells [11]. These observations seem to explain why limited activation of p53 may prolong the lifespan of mice [12] and correlate with observations of age-related decline in p53 function.

In this issue, Leontieva and Blagosklonny further explore the crosstalk between p53 and mTOR in normal cells and highlight the role of the functional status of mTOR in the transition of cells from quiescence to senescence [13]. To separate these activities the authors induced quiescence prior to the induction of p53, using either the DNA-damaging drug etoposide or nutlin-3a, which induces Mdm2-mediated degradation but does not damage DNA. After removal of nutlin-3a and serum refeeding, nutlin-3a-treated cells entered the cell cycle and divided, whereas etoposide-treated cells did not proliferate after etoposide removal and serum addition. However, etoposide-treated cells re-entered cell cycling if co-treated with rapamycin, implying that functional mTOR is essential for permanent loss of proliferative potential. Thus, this study provides evidence that activation of mTOR in quiescent cells is a decision-making factor in the transition to cellular senescence.
